# Skin sympathetic nerve activity: Potential new non‐invasive indicator of exercise metabolic thresholds in adults

**DOI:** 10.14814/phy2.70597

**Published:** 2025-10-05

**Authors:** Ruoxi Wang, Chunxue Tang, Li Li, Lijun Shi

**Affiliations:** ^1^ Department of Exercise Physiology Beijing Sport University Beijing P. R. China; ^2^ Laboratory of Sports Stress and Adaptation of General Administration of Sport Beijing Sport University Beijing China; ^3^ Key Laboratory of Physical Fitness and Exercise, Ministry of Education Beijing Sport University Beijing China

**Keywords:** metabolic thresholds, ramp incremental exercise test, sympathetic nerve activity, ventilatory threshold

## Abstract

This study aims to explore the role of Skin Sympathetic Nerve Activity (SKNA) in exercise monitoring and the determination of metabolic thresholds, and to verify the reliability of the SKNA Thresholds (SKNATs). During the Ramp Incremental Exercise Test (RIET), aSKNA, HR, and gas metabolic parameters were recorded and analyzed to assess their changes and the feasibility of SKNATs. Results showed that, compared to the baseline, aSKNA significantly increased during RIET (*p* < 0.001), and decreased after exercise. The first SKNA threshold (SKNAT_1_) occurred at 43 ± 6% of exercise time, and the second SKNA threshold (SKNAT_2_) occurred at 76 ± 5%. At SKNAT_1_, both HR and aSKNA were significantly lower than at the first ventilatory threshold (VT_1_) (*p* < 0.05, *p* < 0.01), while at SKNAT_2_, all measures except aSKNA were significantly lower than at the second ventilatory threshold (VT_2_) (*p* < 0.05). A strong correlation was observed between SKNATs and VTs, with the highest correlation found at VO_2_ (*r* = 0.902, *p* < 0.0001). ICC showed moderate to strong concordance for all parameters at SKNAT1 and VT1, with higher agreement at the second thresholds. Collectively, aSKNA increases with exercise load during RIET and exhibits non‐linear inflection points. SKNATs show strong potential for predicting VTs, representing a promising emerging indicator for determining metabolic thresholds.

## Graphical abstract

**Skin Sympathetic Nerve Activity as an Exercise Threshold Indicator**. During the incremental load exercise, aSKNA exhibits two distinct inflection points with increasing load intensity, forming SKNATs, a novel metabolic threshold based on SKNA.

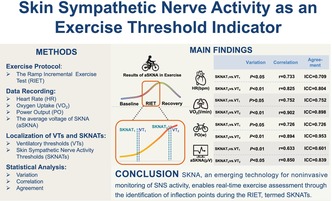

## 1 INTRODUCTION

The exercise metabolic thresholds represent critical physiological inflection points, marking the emergence of non‐linear metabolic changes during the progressive increment of exercise load. Utilizing these thresholds allows for the accurate determination of exercise intensity, thereby enabling the precise prediction of athletic performance, comprehensive monitoring of training status, standardization of exercise routines, identification of risk stratification for clinical interventions, and evaluation of physiological function and treatment outcomes in clinical patients. Therefore, they have been extensively applied in sport training, fitness promotion, and exercise medicine fields (D'Ascenzi et al., 2022; Ferri Marini et al., 2025; Mezzani et al., 2013; Poole et al., 2021; Wolpern et al., 2015).

Currently, the commonly used exercise metabolic thresholds include the lactate thresholds (LTs), the ventilatory thresholds (VTs), and the heart rate variability thresholds (HRVTs). LTs were once regarded as the gold standard for competitive sports training. However, with the publication of cutting‐edge theoretical research such as the lactate shuttle in recent years, LTs still need further practical updates, and the invasive sampling restricts their application in wider popularization (Brooks, 2021; Rossiter, 2021). While VTs have the advantages of non‐invasiveness, standardized measurement, and high correlation with subjective methods such as the talk test, they are suitable for a wider range of populations. Most importantly, VTs have already achieved good research and practical results in the field of exercise and medicine (D'Ascenzi et al., 2022; Jamnick et al., 2020; Keir et al., 2022). Nonetheless, the testing of VTs relies on complex equipment and methodology (Jamnick et al., 2020). In this regard, HRVTs show the advantages of non‐invasiveness and convenience. Based on the HRVTs research, many indicators of HRV can only reflect the overall balance state of the autonomic nervous system (ANS) and cannot reflect the independent level of sympathetic nervous system (SNS) activity (Van Hooren et al., 2025), which may limit its accuracy. It is worth noting that the changes in SNS during exercise are obviously worthy of in‐depth study.

During exercise, the physiological responses of the body involve the coordinated function of multiple systems, particularly the cardiovascular system and the regulation of various tissues and organs, all of which are modulated by the ANS. Within the ANS, the SNS plays a central role in regulating cardiac, vascular, and respiratory functions—especially under high‐intensity conditions. SNS activation enhances muscle excitability, elevates heart rate (HR), and modulates vasomotor tone to optimize blood pressure regulation during exercise, while cooperating with local vasodilatory mechanisms to augment blood flow to engaged skeletal muscles (Fukuta et al., 2024; Wan et al., 2023). Therefore, real‐time monitoring of SNS activity is crucial for accurately assessing exercise load and facilitating personalized adjustments to training intensity. Currently, the most commonly used clinical assessments of SNS activity rely on indirect measurement methods, including neurotransmitters (Grassi et al., 2012; Straub et al., 2000), SNS‐related enzymes, biochemical markers (Schlaich et al., 2007; Schofer et al., 1988), and hemodynamic testing (Fakhrzadeh et al., 2012; Gross et al., 2005). Although these approaches can provide indirect insight into SNS, they are subject to several limitations. Specifically, they often lack the ability to isolate SNS independently and are generally limited in their capacity for real‐time monitoring (Darling et al., 2024). In addition, direct methods for monitoring SNS activity—such as muscle sympathetic nerve activity (MSNA) (White et al., 2015), stellate ganglion nerve activity (SGNA) (Jung et al., 2006; Tan et al., 2008), and subcutaneous nerve activity (SCNA) (Doytchinova et al., 2015; Robinson et al., 2015)—are invasive and pose challenges for long‐term, continuous monitoring, thereby limiting their feasibility in clinical settings. Heart rate variability (HRV), as a noninvasive analytical approach, holds some value in monitoring SNS activity. However, its reliability can be compromised by variability in data acquisition, statistical processing, and potential recording errors, leading to inconsistencies across different datasets.

Recently, a novel method for monitoring SNS activity, known as Skin Sympathetic Nerve Activity (SKNA), has been proposed. This method monitors electrocardiogram signals via external conductive patches, and it has been experimentally demonstrated that the signals processed through electrical filtering originate from the stellate ganglion. The stellate ganglion is one of the cervical sympathetic ganglia. Preganglionic sympathetic fibers synapse in the stellate ganglion and subsequently issue the inferior cardiac nerve, which contributes to the formation of the sympathetic cardiac plexus. This distinguishes SKNA from techniques such as MSNA and SCNA in terms of monitoring principles and recording methodologies. Furthermore, the development of this technology has propelled advancements in research related to the sympathetic nervous system (Kusayama et al., 2020). To date, most studies on SKNA have focused on its use in patients with SNS‐related conditions, such as cardiovascular and renal diseases, where it has been used to assess disease prognosis (Chen et al., 2023; Chen, Meng, et al., 2021; Wang et al., 2023; Zhang et al., 2019; Zhang et al., 2022). However, research exploring the application of SKNA in exercise contexts remains scarce (Liu et al., 2021). Since the physiological principles underlying commonly used methods for determining metabolic thresholds are related to SNS activity, it is important to investigate whether SKNA demonstrates a nonlinear inflection point, similar to other metabolic thresholds, as exercise intensity increases. The objectives of this study are twofold: first, to determine whether Skin Sympathetic Nerve Activity Thresholds (SKNATs) exist during the RIET; and second, the reliability of SKNATs for determining or predicting metabolic thresholds were assessed through reliability analyses with VTs, including correlation, intraclass correlation coefficient (ICC), and Bland–Altman plots.

## 2 MATERIALS AND METHODS

### 2.1 Participants

The participants in this study were healthy adults aged 18–28 years with prior exercise experience. Eligibility criteria included engaging in regular physical activity for at least 1 h per week, achieving a minimum weekly metabolic equivalent (MET) of 600, and being free of sports injuries, cardiovascular disease (CVD) risk factors, chronic illnesses, smoking habits, and long‐term use of pharmaceutical agents.

All participants provided written informed consent before inclusion in the study. The research protocol was approved by the Ethical Review Committee of Exercise Science Experiment at Beijing Sport University (Approval No. 2022149H).

### 2.2 Design

#### 2.2.1 The ramp incremental exercise test (RIET)

The Ramp Incremental Exercise Test (RIET) was conducted using an aerobic power bike dynamometer (Monark 839E, Sweden). Following the Ramp protocol (Sietsema et al., 2021; Wasserman et al., 2012), incremental loads were calculated using the formula proposed by Wasserman et al. (Equation 1). The procedure began with a 5‐min rest period, followed by a 3‐min unloaded warm‐up. Afterward, the RIET commenced with participants pedaling at a constant cadence of 60 rpm. The test continued until exhaustion, at which point participants were instructed to stop immediately and rest for 10 min.

(1) $$\begin{array}{c} \text{Incremental load}\;\left(W/\min\right)=\left[\left(\text{Height}\;\left(\mathrm{cm}\right)-\mathrm{Age}\right)\times 20\;\text{or}\;14\;\text{male or felmale}-\left(150+6\times \text{Weight}\left(\mathrm{kg}\right)\right)\right]/100 \end{array}$$

Exhaustion was determined based on one or more of the following criteria: 1. Inability to maintain a pedaling speed of 60 rpm under a given load, or a sustained drop of 5–10 rpm below 60 rpm for more than 5 s. 2. A respiratory quotient (RQ) of ≥1.2. 3. Participant discomfort leading to voluntary termination of the test.

SKNA, power output (PO), HR, and gas metabolic indicators such as oxygen uptake (VO_2_) and carbon dioxide output (VCO_2_) were continuously monitored throughout the test.

#### 2.2.2 Data recording

**FIGURE 1:**
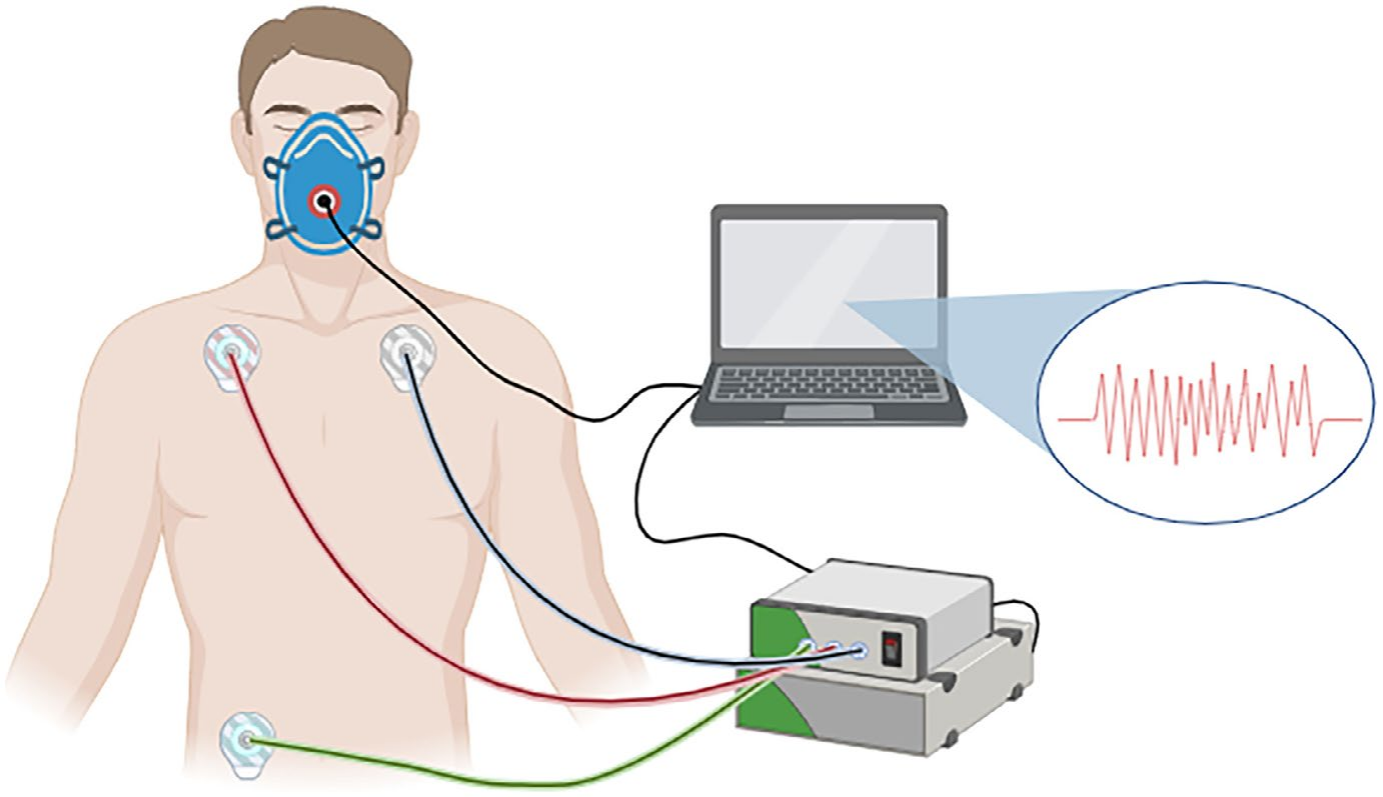
Device Connections. The three curves connected to the participants' torso represent the SKNA lead lines, which are linked to the bioelectric acquisition system interfaced with a computer. The curve connected to the respiratory mask represents the VO_2_ acquisition line, which is also connected to the computer. Figure created in https://BioRender.com.

SKNA was recorded using a bioelectric acquisition system (AD Instruments). Standard electrocardiographic electrodes were connected to lead wires and affixed to the designated positions while participants were in a cycling position (Figure 1). Before starting the test, the acquisition software was activated to verify signal stability. The test commenced when the average voltage fluctuations stabilized above and below 1 μV. The SKNA acquisition software was configured to sample signals at a rate of 10,000 samples per second, using an input range of ±5 mV. A bandpass filter (500–1000 Hz) was applied to display raw SKNA data. The primary SKNA index analyzed in this study was the average voltage of SKNA (aSKNA, μV). The aSKNA channel was programmed to calculate the total voltage divided by the number of samples within a given window, providing a sliding average over 5 s intervals. The data output interval was set to 1 s.

Gas exchange and heart rate (HR) data were collected using a high‐resolution gas metabolism system (Cortex MetaLyzer® 3BR2, Germany) and a compatible heart rate monitor. The supporting software (Cortex MetaSoft®, Germany) was used for data storage and processing. Daily calibration of the gas metabolism system, including equipment warm‐up, barometric pressure calibration, gas calibration, and volumetric calibration, was performed following the manufacturer's guidelines.

#### 2.2.3 Localization of VTs and SKNATs

Ventilatory thresholds (VTs) were identified as described in previous research (Keir et al., 2022). To confirm and localize SKNATs, an objective mathematical‐based method (Algorithm) and a subjective visual inspection based on biological interpretation (Physiological Principles) were jointly employed. Firstly, by plotting aSKNA against time, the curve revealed an overall upward trend, with two inflection points where the increase trend changes. After these points, the curve continues to rise, and 1–3 time points that meet this condition are selected as possible options. Subsequently, the dynamic programming algorithm from the Python library Rupture—designed for analyzing non‐stationary signals and detecting change points—was applied to analyze the curve. The algorithm was configured to identify 2–7 change points, from which those deviating within 30 s of candidate time points were selected as the definitive thresholds. If the algorithm‐determined point exceeded this 30 s window, visual judgment was used to confirm the curve's trend before and after the inflection. Beyond these inflection points, the curve continued to rise steadily in a linear manner. Finally, for each individual, 2 time points meeting these criteria were identified. The first identified inflection point was defined as the first SKNA threshold (SKNAT_1_), and the second inflection point was defined as the second SKNA threshold (SKNAT_2_).

aSKNA, VO_2_, HR, and PO were primary parameters used to compare VTs and SKNATs. VO_2_ and HR were used to monitor changes in participants' internal load, while PO was for external load throughout the test.

#### 2.2.4 Statistical analysis

Statistical analyses were conducted using SPSS Statistics 26, with results reported as mean ± standard deviation (SD). Graphical representations were created using GraphPad Prism 8. Statistical tests included one‐way repeated measures ANOVA, paired *t*‐tests, and Pearson's correlation coefficient (non‐parametric correlations were analyzed using Spearman's rank correlation coefficient). ICC was calculated to evaluate the consistency of VTs and SKNATs, with thresholds defined as follows: 0.5–0.75 for moderate consistency, 0.75–0.9 for high consistency, and >0.9 for very high consistency. Bland–Altman plots were also generated to assess agreement.

## 3 RESULTS

### 3.1 Participants

The demographic and baseline characteristics of the 22 participants who completed the incremental loading exercise experiment are presented in Table 1. The cohort included 13 males and 9 females.

**TABLE 1:** Participants' information.

	Male (*n* = 13)	Female (*n* = 9)	Total (*n* = 22)
Age (years)	21.7 ± 2.9	23.8 ± 2.0	22.5 ± 2.7
Height (cm)	178.0 ± 5.3	161.2 ± 8.2	171.1 ± 10.6
Weight (kg)	73.5 ± 7.7	55.3 ± 7.2	66.1 ± 11.7
BMI (kg/m^2^)	23.2 ± 1.8	21.2 ± 1.5	22.4 ± 2.0
Body fat percentage %	20.8% ± 6%	30.3% ± 4%	24.7% ± 7%
Week's metabolic (MET‐min)	3221.5 ± 1122.4	3412.8 ± 1530.5	3299.8 ± 1273.4

#### 3.1.1 aSKNA's patterns change in RIET

**FIGURE 2:**
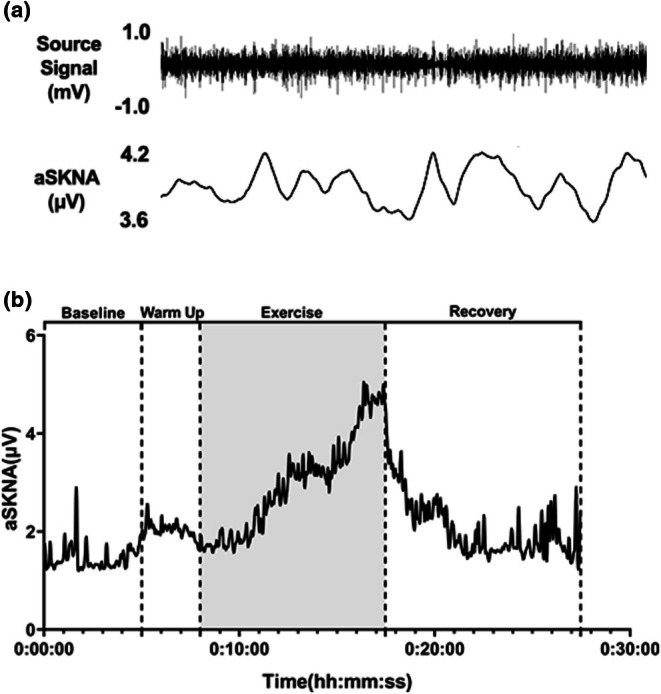
(a) A segment of the raw SKNA recording. (b) Trends in aSKNA changes in a participant's experiment. Due to variations in exercise duration, only one participant was selected to represent the complete curve as an example.

The duration of RIET showed a high degree of consistency across participants, with an average time of 568 ± 101 s under the applied protocol. The original SKNA recording and the trends in aSKNA during RIET are illustrated in Figure 2, demonstrating an upward trajectory with incremental loading and peaking at the conclusion of the exercise period. The relative increase in aSKNA during exercise was quantified using the rate of aSKNA increase (aSKNArate, Equation 2), the results revealed an approximate twofold increase in peak aSKNA during exercise compared to the resting phase (2.17 ± 0.59).

(2) $$\text{aSKNArate}=\frac{\text{aSKNAmax}-\text{aSKNAbase}}{\text{aSKNAbase}}$$

Furthermore, the peak aSKNA during exercise was significantly elevated compared to the resting state (5.51 ± 0.99 μV vs. 1.79 ± 0.48 μV, *p* < 0.001). Following exercise, aSKNA exhibited a marked decline. After 10 min of recovery, the decrease from the exercise peak was significant (5.51 ± 0.99 μV vs. 2.16 ± 0.46 μV, *p* < 0.001) and remained distinct from the resting value (*p* = 0.001 < 0.01).

**FIGURE 3:**
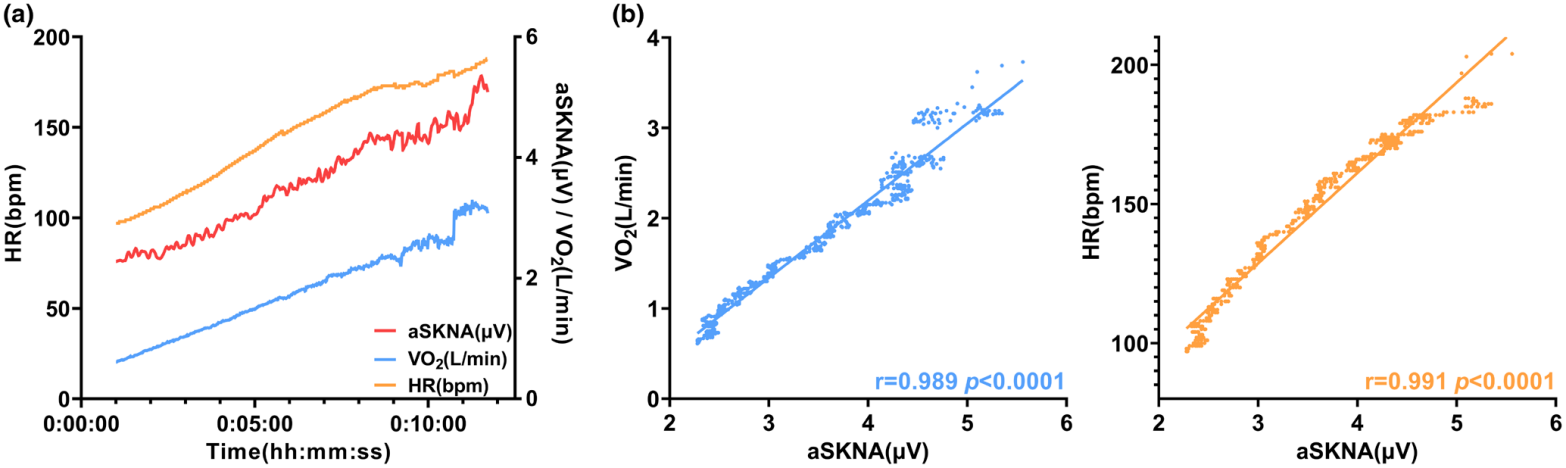
Trends and correlations in aSKNA, VO_2_, and HR during exercise. (a) Trend profiles in aSKNA, VO_2_, and HR during RIET. (b) Correlation analysis results for aSKNA with VO_2_ and HR.

In addition, as depicted in Figure 3, the tendency of aSKNA changes paralleled those of VO_2_ and HR during exercise, demonstrating strong and statistically significant correlations (r = 0.991, *p* < 0.0001; r = 0.989, *p* < 0.0001).

### 3.2 Results of localization of SKNATs in RIET

**FIGURE 4:**
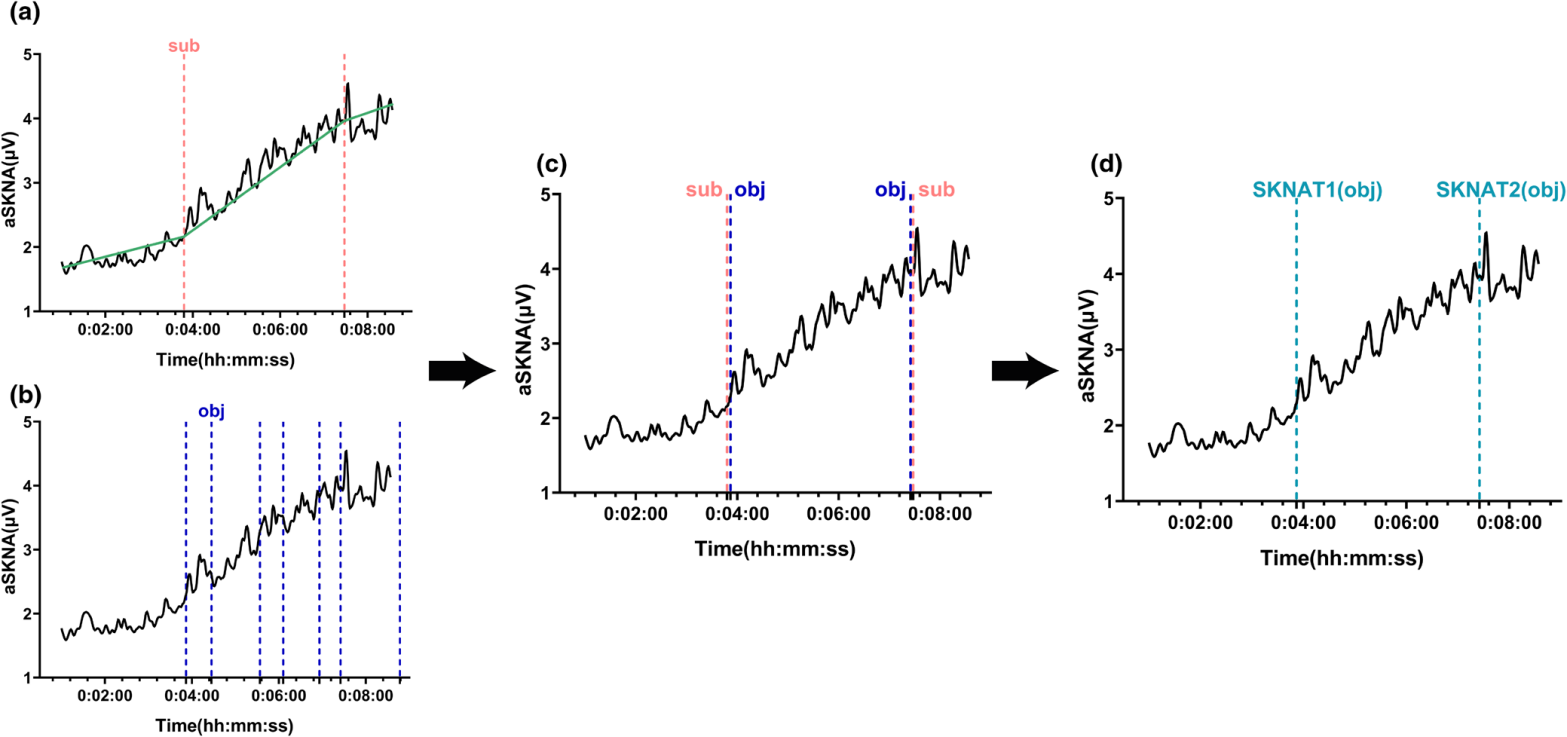
Localization of SKNATs. SKNAT‐sub refers to the inflection point identified through the visual judgment method, SKNATs‐obj refers to the inflection point determined using the dynamic programming algorithm. (a) The inflection point identified via the visual judgment method. (b) The inflection point determined through the dynamic programming algorithm. (c) The overlap of the two methods. (d) The SKNAT identified by the dynamic programming algorithm.

After analyzing each participant's aSKNA profile using the threshold determination method, two distinct threshold points were identified. Figure 4 provides a schematic representation of the threshold determination process. Both objective, mathematically based method and subjective, biology‐interpretation visual inspection based method were employed to identify several potential points. Upon combining the results of both approaches, two points were identified that coincided within a 30 s difference and aligned with the physiological changes observed during RIET in humans. Consequently, the algorithmic results were taken as the final determination of the SKNATs, which were defined as SKNAT_1_ and SKNAT_2_, respectively. The appearance of SKNAT_1_ marks the end of a relatively stable period of aSKNA, after which aSKNA begins a systematic rise. Following SKNAT_2_, the rise in aSKNA significantly differs from the previous phase, reaching its peak within this period.

The relative positions of the threshold points were further analyzed, and the results are presented in Table 2. SKNAT_1_ generally appeared in the early–middle stage of the incremental exercise protocol (43 ± 6% of total exercise time), preceding VT_1_ (48 ± 10% of total exercise time). In contrast, SKNAT_2_ typically emerged during the late stage of exercise (76 ± 5% of total exercise time) and appeared earlier than VT_2_ (82 ± 7% of total exercise time).

**TABLE 2:** SKNATs versus VTs time results.

		% of exercise time
SKNATs	SKNAT_1_	43 ± 6
	SKNAT_2_	76 ± 5
VTs	VT_1_	48 ± 10
	VT_2_	82 ± 7

*Note*: % Total time represents the proportion of the total exercise duration at which the threshold point occurs.

### 3.3 SKNATs vs. VTs

#### 3.3.1 Variation in SKNATs vs. VTs

**FIGURE 5:**
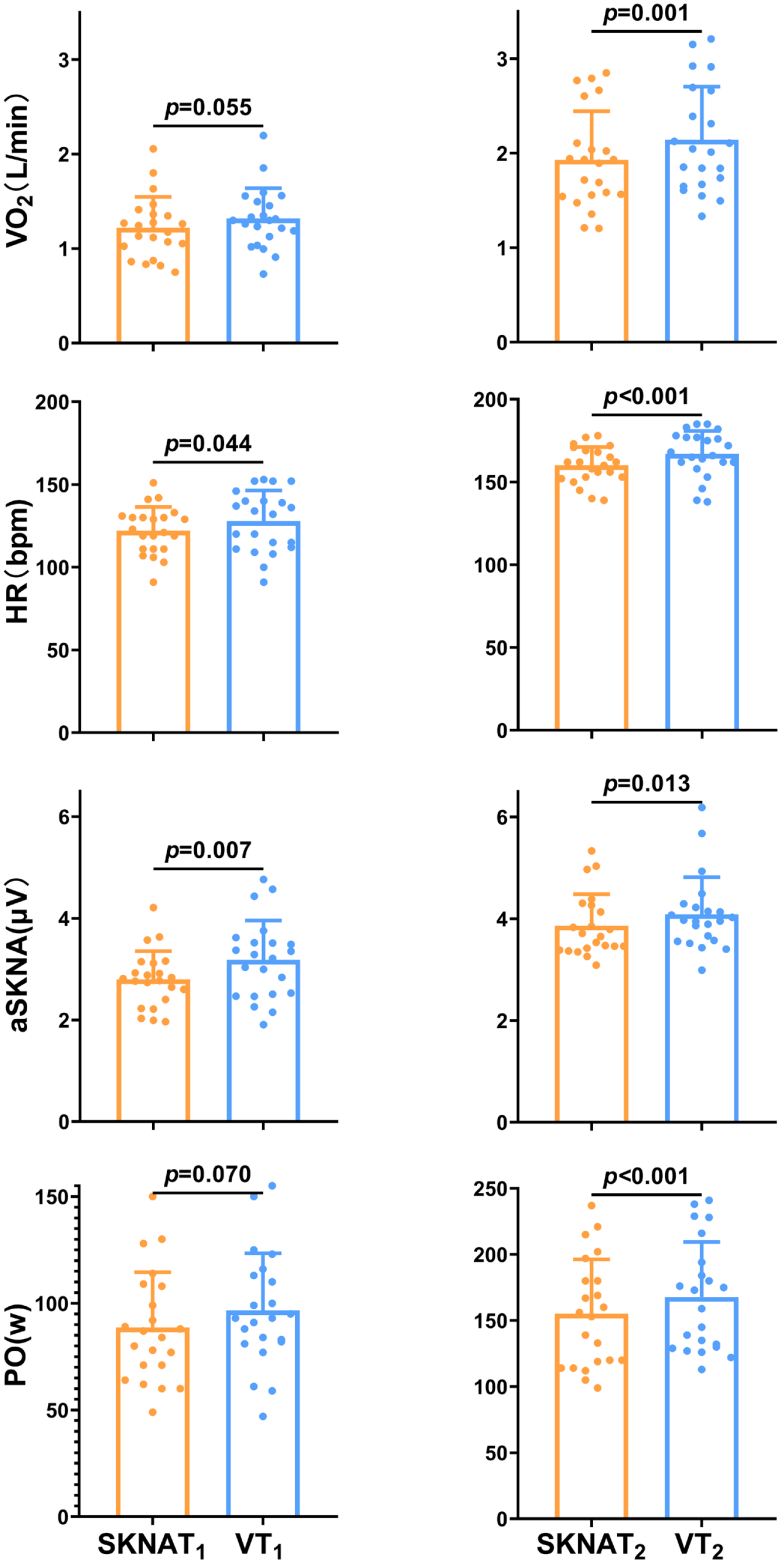
Variation in SKNATs versus VTs.

The corresponding indicator values at SKNATs and VTs time points are summarized in Table 3. The mean values of all indicators at SKNATs were consistently lower than those at VTs. According to Figure 5, no significant difference in VO_2_ was observed between SKNAT_1_ and VT_1_ (*p* = 0.055 >0.05). However, HR at SKNAT_1_ was significantly lower than at VT_1_ (*p* = 0.044 <0.05), and aSKNA was markedly lower at SKNAT_1_ compared to VT_1_ (*p* = 0.007 <0.01). There was no significant difference in PO between SKNAT_1_ and VT_1_ (*p* = 0.070 >0.05). For SKNAT_2_ and VT_2_, significant differences were observed across all key indicators. VO_2_, HR, and PO were considerably lower at SKNAT_2_ compared to VT_2_ (*p* = 0.001 <0.01, *p* < 0.001, *p* < 0.001, *p* < 0.001). Similarly, aSKNA at SKNAT_2_ was significantly lower than at VT_2_ (*p* = 0.013 <0.05).

**TABLE 3:** SKNATs and VTs correspondence indicators.

	SKNAT_1_	VT_1_	SKNAT_2_	VT_2_
VO_2_ (L/min)	1.22 ± 0.33	1.32 ± 0.32	1.93 ± 0.51	2.14 ± 0.56
HR (bpm)	122.13 ± 14.29	127.90 ± 18.53	160.05 ± 11.11	166.96 ± 13.89
PO (w)	88.64 ± 25.89	96.60 ± 26.79	155.10 ± 41.18	166 (130.5–191.5)
aSKNA (μV)	2.80 ± 0.56	3.18 ± 0.77	3.87 ± 0.62	4.09 ± 0.73

*Note*: PO is calculated using the formula for this movement time.

#### 3.3.2 Correlations in SKNATs and VTs

**FIGURE 6:**
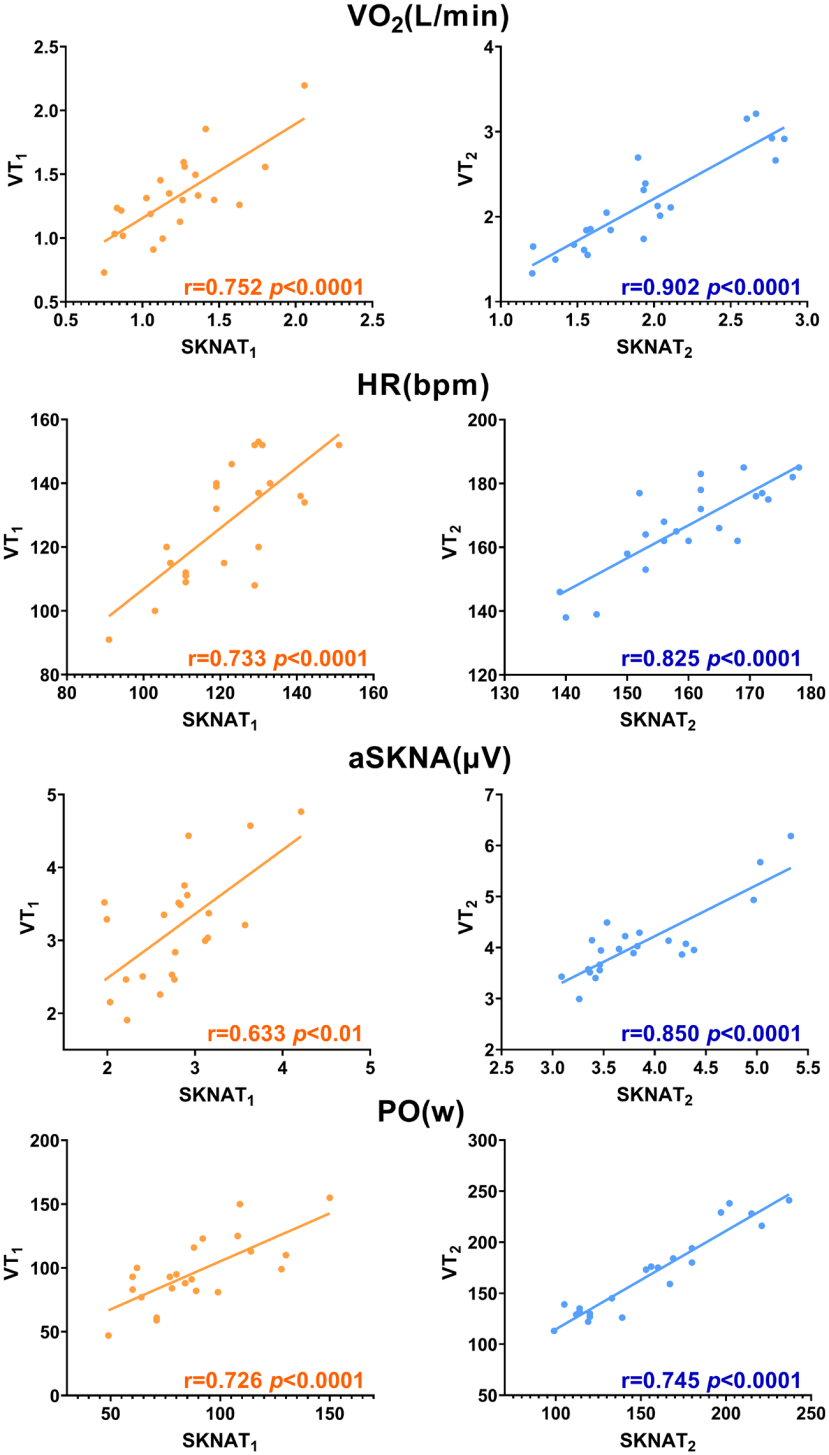
Correlations in SKNATs and VTs. *r* > 0.6 is considered indicative of a strong correlation.

SKNAT_1_ and VT_1_ demonstrated strong correlations across various indicators, with VO_2_ exhibiting the highest correlation coefficient (*r* = 0.752, *p* < 0.0001). The correlation for aSKNA was relatively lower but remained significant (*r* = 0.633, *p* < 0.001). As shown in Figure 6, the correlation coefficients between SKNAT_2_ and VT_2_ indicators were notably higher compared to those between SKNAT_1_ and VT_1_, indicating a closer alignment between SKNAT_2_ and VT_2_.

#### 3.3.3 Agreement in SKNATs vs. VTs

According to Table 4, ICC values for VO_2_, HR, and PO under SKNAT_1_ were high, with PO demonstrating the greatest consistency with VT_1_. Conversely, in the SKNAT_2_ versus VT_2_ comparison, PO exhibited the highest ICC value, followed closely by VO_2_, HR, and aSKNA. All ICC values in the SKNAT_2_ vs. VT_2_ analysis surpassed those observed in the SKNAT_1_ versus VT_1_ comparison.

**TABLE 4:** ICC results in SKNATs versus VTs.

		VO_2_ (L/min)	HR (bpm)	aSKNA (μV)	PO (w)
ICC	SKNAT_1_vs. VT_1_	0.752	0.709	0.601	0.726
	SKNAT_2_vs. VT_2_	0.898	0.804	0.839	0.953

**FIGURE 7:**
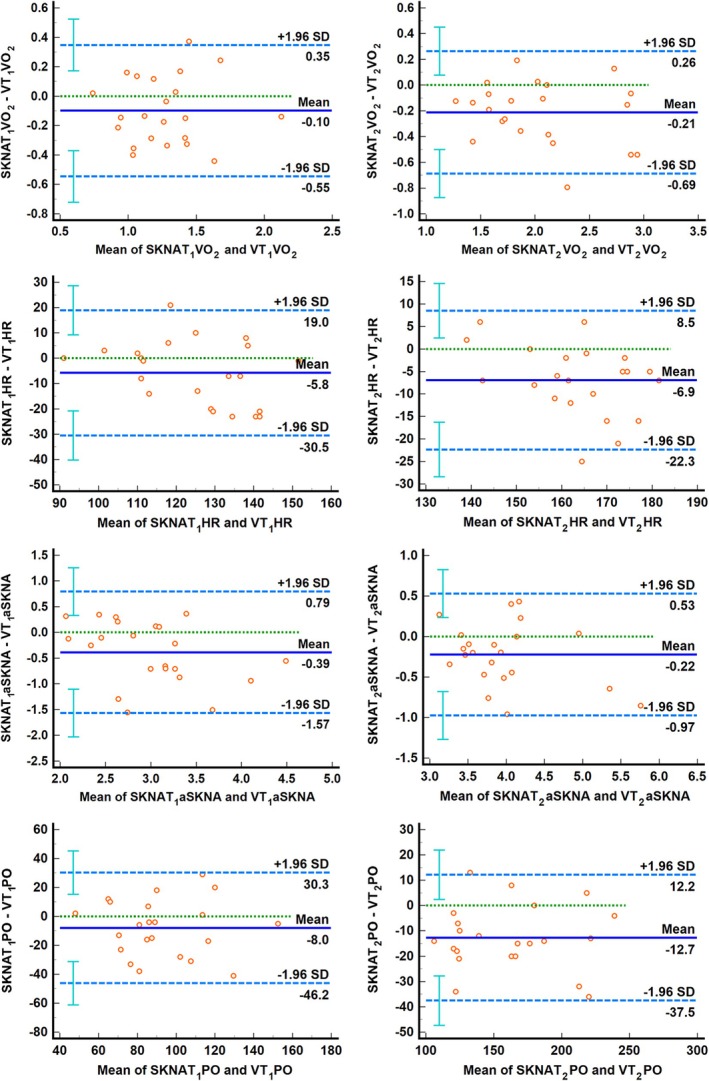
Bland–Altman plot of SKNATs versus VTs. The dashed lines at the top and bottom of the figure represent the mean difference between the measurements of the two methods, while the dashed line in the middle indicates the 95% limits of agreement (+1.96 SD and −1.96 SD). The points on the figure represent the differences between the measured values of individual samples and the mean value. Ideally, most of these points should fall within the 95% limits of agreement.

Figure 7 shows the Brand‐Altman plot for SKNATs and VTs. As the figure shows, in the comparisons of VO_2_, HR, and PO (SKNAT_2_ vs. VT_2_), there is one data point (4.5%) that falls outside the limits of agreement (LoA), but all fall within the 95% LoA range. All other indicators fall entirely within the LoA.

## 4 DISCUSSION

This study presents several notable findings: (1) aSKNA during RIET fluctuates with exercise intensity, increasing with incremental loading and rapidly decreasing from its peak at the conclusion of exercise. (2) Two distinct thresholds of aSKNA, identified as SKNAT_1_ and SKNAT_2_, were observed. (3) While the indexes associated with SKNATs and VTs differ, they exhibit significant correlations and high consistency.

### 4.1 aSKNA may serve as a biomarker for exercise monitoring

During physical exertion, SNS plays a pivotal role in modulating cardiovascular, hormonal, and metabolic responses by perceiving and responding to exercise intensity. This modulation includes stimulating the release of epinephrine from the adrenal medulla via SNS activation, which subsequently leads to increased heart rate and enhanced ventricular contraction (Fisher et al., 2015). Previous studies have demonstrated that SKNA can be employed to monitor changes in aSKNA during exercise in individuals with various comorbidities, suggesting its potential as a fitness biomarker (Liu et al., 2021). In this study, it was further confirmed that aSKNA is strongly correlated with VO_2_ and HR during exercise (Figure 3). In contrast to prior findings, our study highlights the significance of aSKNA in monitoring exercise intensity within a healthy population. Furthermore, the phenomenon of increased tension in the vagus nerve at the conclusion of the exercise period, which even exceeds the pre‐exercise level, is known as vagal reactivation. Consequently, aSKNA may be at a lower level than the baseline in the recovery phase after exercise. However, in this study, aSKNA failed to revert to the resting level post‐exercise, which indicates that a longer duration than 10 min may be required for the ANS to be restored to its baseline after extreme physical exertion.

In summary, aSKNA demonstrates a high degree of sensitivity to exercise responses, effectively distinguishing between the various phases of exercise and recovery. This underscores its potential as a valuable tool for assessing physiological adaptations during RIET.

### 4.2 aSKNA exhibits metabolic thresholds during the RIET

An analysis of the aSKNA curve revealed three distinct phases during the rise in aSKNA, marked by two inflection points where the rate of increase in aSKNA shifted. These inflection points are identified as aSKNA thresholds (Figure 4).

In this study, SKNAT_1_ was observed during the early phase of exercise, reflecting the transition in SNS excitability following the onset of physical activity. During rest, the Parasympathetic Nervous System (PNS) predominantly governs autonomic function. As exercise begins and the load gradually increases, central commands from the cerebral cortex stimulate the medullary cardiovascular control center, affecting arterial baroreflex suppression and reducing PNS activity, that is, the withdrawal of the PNS, thereby triggering a rapid increase in HR. Simultaneously, rapid feedback from muscle mechanoreceptors induces suppression of PNS during the initial phase of exercise (Michael et al., 2017). In parallel, SNS activity progressively intensifies, regulating HR, cardiac output, and vascular tone. This ensures optimized blood flow to active tissues while restricting circulation to non‐exercising systems. Previous research on HRVTs has suggested that the emergence of HRVT_1_ correlates with the decline in PNS activity as exercise load increases (Michael et al., 2017). Therefore, based on the physiological mechanisms of the SNS discussed above, it is thought that there is an inflection point representing increased SNS, as indicated by aSKNA, during the early exercise phase, that is, SKNAT_1_.

Based on the results, SKNAT_2_ occurs during the middle to late stages of exercise, indicating a divergence in the trend of SNS activity escalation compared to earlier stages. This inflection point is closely linked to the physiological mechanisms underlying VTs. VTs are commonly understood to arise as exercise intensity increases, prompting a metabolic shift toward anaerobic pathways. This shift leads to the accumulation of lactic acid and an associated rise in hydrogen ions (H^+^). The excess H^+^ interacts with bicarbonate, resulting in the release of significant amounts of CO_2_, which stimulates ventilation in response to elevated blood CO_2_ levels. Sensory neurons in the carotid artery and aortic body detect changes in blood pH, triggering chemoreflexes that suppress PNS activity while amplifying SNS activity (Wan et al., 2023). Moreover, at higher exercise intensities, the respiratory muscles—one of the triggers of the exercise pressor reflex—are progressively activated. Their elevated metabolic demand enhances mechanical and chemical stimuli within these muscles, thereby inducing the reflex that further promotes the enhancement of SNS activity (Chen, Sun, et al., 2021; Keir et al., 2024; Michael et al., 2017).

In summary, aSKNA not only monitors prolonged incremental exercise in healthy individuals but also identifies two distinct metabolic thresholds, namely SKNAT_1_ and SKNAT_2_, which reflect the dynamic changes in SNS activity in response to progressively increasing exercise intensity.

### 4.3 SKNATs are associated with VTs

As shown in Table 2, aSKNA thresholds appeared earlier than VTs during RIET. This temporal difference can be attributed to the influence of SNS activity on respiratory rhythm. With increasing exercise load, the SNS becomes more excited, HR accelerates, and the oxygen demand increases, leading to faster breathing and deeper ventilation, causing aSKNA changes to precede the corresponding increases in VO_2_.

The comparison between SKNAT_1_ and VT_1_ revealed similarity in VO_2_ and PO results, while SKNAT_2_ and VT_2_ showed significant differences across all measured indicators (Figure 5). Both HR and aSKNA demonstrated variations, which may be attributed to their distinct responses. HR, as a direct indicator of exercise intensity, is highly sensitive to changes in workload, while aSKNA directly reflects changes in the SNS, which is highly correlated with HR. Therefore, under different exercise times, HR and aSKNA exhibit differences in their performance. The discrepancies in ventilation‐related indicators between SKNAT_2_ and VT_2_ may be explained by the respiratory compensatory point (RCP). During exercise, ventilation (VE) generally increases in proportion to VCO_2_. However, at the RCP, VE exceeds VCO_2_, leading to hyperventilation and a nonlinear increase in ventilation (Keir et al., 2024). In this study, SKNAT_2_ occurred earlier than VT_2_, suggesting that the RCP may not have been reached by the time SKNAT_2_ was identified. Consequently, VO_2_ at SKNAT_2_ was significantly lower than at VT_2_.

A strong correlation and substantial consistency were observed across all indicators between SKNATs and VTs (Figures 6, 7). The results indicate that although SKNATs do not temporally coincide with VTs and certain discrepancies exist in specific indicators, the internal and external load parameters at the corresponding time points exhibit strong consistency with VTs. This suggests that although SNS may be affected by psychological fluctuations or environmental factors prior to the start of exercise—thereby influencing respiratory rhythm and other physiological functions—during RIET, the regulation of the SNS is not independent. Instead, the SNS functions in close coordination with the respiratory and other physiological systems, collectively adapting to meet the metabolic demands imposed by RIET.

In conclusion, the results indicate that SKNATs hold potential as a reliable method for determining metabolic thresholds, with SKNAT_2_ showing higher feasibility and accuracy than SKNAT_1_.

### 4.4 Practical applications

The metabolic threshold serves as a critical parameter for determining exercise intensity and designing effective exercise prescriptions. Compared to LT and VTs, SKNATs offer a more convenient solution. Data analysis reveals strong correlations and high consistency between SKNATs and corresponding VTs metrics, enabling SKNATs to complement VTs in developing personalized training plans and assessing exercise capacity. The SKNA technique detects sympathetic nerve activity through skin‐surface electrodes, providing a real‐time reflection of SNS activation. During exercise, SNS activation serves as a core driver of various physiological responses, including increased HR and altered breathing rhythms. Thus, by directly capturing changes in SNS activity, SKNATs offer more granular data for evaluating exercise capacity and formulating exercise prescriptions. In practice, exercise intensities approaching SKNAT_2_ may be targeted to enhance aerobic metabolism and endurance, with subsequent re‐evaluation of the temporal shift in SKNAT_2_ occurrence after the training cycle to assess the efficacy of the training protocol. However, it should be noted that the determination and application of SKNATs remain in a preliminary exploratory stage, and they cannot yet be utilized as a standalone indicator or a direct replacement for conventional methods. Moreover, with the widespread application of SKNA technology in both clinical and healthy populations, and the continuous optimization of recording devices, the feasibility of using SKNA to monitor SNS and determine metabolic thresholds during exercise has been further improved. For example, recent studies have applied a Holter‐like neuECG recorder for SKNA acquisition, which enables continuous data collection for up to 8 h and features a compact design (30 × 25 × 5 mm) (Chen, Sun, et al., 2021), offering greater portability compared to the original recording equipment.

### 4.5 Limitations

This study hypothesized that a threshold point for SKNA monitoring exists in RIET, in alignment with physiological principles. To investigate this, a potential method for determining the threshold was explored. Additionally, constrained by the limitations of the SKNA equipment, it is currently impossible to monitor SNS‐associated changes in body temperature. The thermoregulatory mechanisms underlying the generation of SKNATs have not yet been analyzed, and other physiological principles not discussed in this study also require further elaboration. However, the findings suggest that more rigorous approaches could be developed to identify SKNATs with greater precision. As research into SKNA applications continues to expand, the number of parametric indicators for SKNA has progressively increased, leading to a more refined and mature parameter system. While this study focused solely on aSKNA, these emerging parametric indicators may offer valuable insights for identifying additional anaerobic thresholds. In addition, the bioelectric acquisition system is the most commonly used device in SKNA technology. However, these devices are typically large, heavy, and inconvenient to carry, and their connection cables are limited in length. This restricts the freedom of movement of the experimental environment and test subjects during long‐term continuous measurement and monitoring, which in turn affects the application and development of this technology in fields other than disease research.

It is important to note that the study population consisted exclusively of healthy individuals with exercise experience. The applicability and feasibility of SKNATs in populations with underlying health conditions remain areas for future investigation.

## 5 CONCLUSIONS

SKNA enables real‐time monitoring of exercise responses in healthy individuals. Furthermore, SKNATs demonstrate reliability in predicting VTs and exhibit potential as a novel metabolic threshold indicator.

## AUTHOR CONTRIBUTIONS

Wang and Tang collected the data, analyzed the data, and wrote the manuscript. Shi and Li reviewed and edited the initial draft. Shi supervised the project, administered the project, and performed conceptualization.

## FUNDING INFORMATION

This work was supported by the National Key R&D Program (grant number 2022YFC3600201), the Fundamental Research Funds for the Central Universities (2025KYPT03 and 2024JCYJ001).

## Data Availability

The data presented in this study can be obtained from the corresponding author. These data are not currently available to the public because of the privacy and confidentiality of the participants.
